# Effects of three equine-related training modalities on inhibitory control in children aged 7–8 years: an exploratory randomized controlled trial

**DOI:** 10.3389/fpsyg.2026.1752523

**Published:** 2026-05-29

**Authors:** Xiaodong Cheng, Naichun Ji, Cheng Chen

**Affiliations:** 1 Xi’an Medical University, Physical Education Department, Blockchain and Healthcare, Health Service Research Center, Xi'an, China; 2 Guangzhou Medical University, Physical Education Department, Guangdong, China

**Keywords:** children, flanker task, fNIRS, inhibitory control, equine-related training

## Abstract

**Introduction:**

Horseback riding training (HRT), horseback riding simulator training (HRST), and horse companion training (HCT) can improve physical fitness, cognitive abilities, and emotional well-being. However, their effects on inhibitory control (IC) are not well studied.

**Methods:**

This exploratory randomized controlled trial examined the effects of HRT, HRST, and HCT on IC in children aged 7-8 years and analyzed brain neural mechanisms using functional near-infrared spectroscopy (fNIRS). Twenty-four children aged 7-8 years from Maple Leaf International School-Xi’an were randomly divided into three groups: the horseback riding group (HRTG), the horseback riding simulator group (HRSG), and the horse companion group (HCG), with 8 children in each group. The 24 subjects received training with HRT, HRST, and HCT. Each group received its assigned intervention: the HRTG received HRT, the HRSG received HRST and the HCG received HCT. All subjects performed the Flanker task while fNIRS data were collected.

**Results:**

After 12 weeks of training, the HRTG showed faster reaction times and higher accuracy (*p* < 0.01) in both congruent and incongruent Flanker tasks. Importantly, fNIRS data showed that HRTG significantly increased the mean change in Oxy-Hb in channels 7, 21, and 23, which are approximately over the right lateral prefrontal cortex (putative R-DLPFC region) during the congruent Flanker task.

**Conclusion:**

HRT may improve IC and activate the R-DLPFC in children aged 7-8 years.

## 1 Introduction

In cognitive developmental psychology, inhibitory control (IC) refers to the voluntary control or inhibition of target-unrelated stimuli, cognitive responses, and behavioral responses (Nigg, 2000). IC is a higher cognitive function. The greater an individual’s cognitive abilities, the faster and more accurately they can respond to important information, resulting in more efficient memory encoding and better academic performance (Vock et al., 2011). IC is also closely related to language comprehension, writing ability, mathematical operations (Titz and Karbach, 2014), and sports performance (Albaladejo-García et al., 2023). Furthermore, IC plays a crucial role in an individual’s life, especially during childhood when it develops rapidly. At this stage, the improvement of reaction time and accuracy is of great significance to cognitive growth. However, as people age, IC tends to decline (Bedard et al., 2002), which highlights the importance of promoting its development in early life stages to support lifelong cognitive function.

Existing studies use several paradigmatic tasks to evaluate IC. One of these tasks1 is the Flanker task (Eriksen and Eriksen, 1974), which assesses resistance to interference using arrays of letters, arrows, or shapes. The Go/NoGo task (Logan et al., 1997) examines response inhibition by requiring participants to respond to some stimuli but not others. Another related task is the stop-signal task, where a response must be halted after initiation upon receiving a stop signal. Another task is the color/word Stroop task (Stroop, 1935), which involves color words printed in inconsistent ink colors (e.g., the word “red” printed in green ink). Participants must name the ink color rather than read the word, requiring suppression of the automatic reading process. Because color naming and word reading are separate processes, their inconsistency requires cognitive flexibility, as one process interferes with the other.

Existing studies using functional magnetic resonance imaging (fMRI) to explore IC in children have limitations, including difficulty for children to remain still and the limited spatial resolution of EEG (Zhou et al., 2022). Therefore, questions about the neural basis of these processes and their development remain. The functional near-infrared spectroscopy (fNIRS) is quieter and less sensitive to motion than fMRI, allowing children to be tested while awake in a non-isolated environment. Thus, it is a viable tool for these studies.

In a study that used the Flanker task to assess IC, it was shown that their ability was improved by motor interventions. Cheng et al. (2023) reported that horseback riding training (HRT) was associated with improved IC in children by enhancing their cognitive and motor coordination abilities, which is essential for controlling attention and inhibiting irrelevant stimuli. Schroeder (2015) similarly used a horseback riding intervention, which has also been shown to be effective in improving children’s IC. However, given the exploratory nature and small sample size of this study, the potential effects of HRT on IC should be interpreted with caution. These findings provide preliminary support for further investigation into whether HRT is associated with IC changes and associated neural activation in children.

Horseback riding simulator training (HRST) has been shown to have similar effects on IC as HRT, although the extent of this similarity depends on various factors such as the intensity of the training and the type of motor coordination involved. Previous studies have shown that HRST has a positive impact on physical muscle strength, gross motor coordination and postural improvement in children (Borges et al., 2011). HRST reproduces some of the basic movements of the horse, and the physics of the riding simulator is to some extent similar to that of a real horse, which makes it effective for riders to train on a riding simulator to promote muscle activation and postural adjustments (McGibbon et al., 2009), and these repetitions strengthen pelvic and lumbar muscle strength and improve postural balance and control of the trunk (Shinomiya et al., 2003). In addition, Mitani et al. (2008) found that 12 weeks of HRST increased trunk utilization efficiency, improved coordinated trunk and leg movements and strengthened leg function. However, there has been relatively little research on cognition in HRST. Accumulating evidence suggests that physical, cognitive and affective correspond to each other and contribute to each other. Relevant studies have shown that horse companion training (HCT) can effectively improve children’s emotions and reduce emotional disorders such as anxiety, depression and hyperactivity (Maresca et al., 2022). But it is also poorly known whether it can improve cognitive development.

Overall, the use of three different training modalities to assess IC using the Flanker task paradigm and the fNIRS technique to study the changes in neural mechanisms of the brain has certain research value.

## 2 Methods

### 2.1 Subjects and study design

#### 2.1.1 Subjects

In this experiment, the sample size was calculated using G*Power software (version 3.1.9.7; Franz Faul, University Kiel, Germany) (Faul et al., 2007). The parameters were set as follows: *α* = 0.05, power = 0.80, effect size = 0.35, statistical test = repeated measures, with-between interaction, number of groups = 3, number of measurements = 2. Based on the above conditions, a sample size of 8 subjects per group was determined, so a total of 24 subjects were selected. A total of 24 male and female children aged 7–8 years old from Maple Leaf International School-Xi’an were selected as test subjects and divided into horseback riding group (HRTG), horseback riding simulator group (HRSG) and horse companion group (HCG), with 8 participants in each group, 4 boys and girls, each aged 7 and 8 years, and associated training interventions. Participants were randomly assigned to the HRTG, HRSG, or HCG groups in a 1:1:1 ratio. Randomization sequences were generated by a researcher who was not involved in the outcome assessment before subject enrollment using the Microsoft Excel RAND function. To ensure allocation concealment, group allocation results were placed in sequentially numbered opaque envelopes and revealed only after baseline assessment was completed. Due to the limited nature of the intervention, participants and intervention implementers were unable to achieve blinding; however, the outcome assessors responsible for performing the Flanker task and the technicians responsible for fNIRS acquisition did not participate in group assignment. In addition, all datasets were de-identified through subject coding, and group labels were masked during the data preprocessing and statistical analysis stages, so that data analysts remained blind to group identity until the main analysis was completed. The basic information of 24 children aged 7–8 years was shown in Table 1. The institutional ethics committee of the Capital University of Physical Education and Sports China approved all procedures and protocols (No. 2021A44). All subjects obtained informed consent in the presence of their parents. In addition, all procedures in this study followed the principles set out in the Declaration of Helsinki.

**Table 1:** Basic subject information.

Measures	HRTG (*N* = 8)	HRSG (*N* = 8)	HCG (*N* = 8)
Years	7.5	7.5	7.5
Height (cm)	128.50 ± 7.35	128.13 ± 5.82	127.38 ± 5.10
Weight (kg)	27.63 ± 2.82	26.96 ± 3.53	27.16 ± 2.27
BMI (kg/m^2^)	16.73 ± 1.16	16.36 ± 1.08	16.72 ± 0.61

The inclusion and exclusion criteria were as follows: (1) Inclusion criteria:(1) The children’s ages are 7 to 8 years old; (2) the children can complete the Flanker task and the fNIRS test; (3) children can participate in a 12-week intervention; (4) the children had no formal HRT experience before. (2) Exclusion Criteria: (1) Children with neurodevelopmental disorders are excluded; (2) children with visual or motor impairments are excluded; (3) children currently using drugs that may affect cognitive or motor function are excluded; (4) children who were unable to complete the baseline test were excluded.

#### 2.1.2 Research design

**Figure 1:**
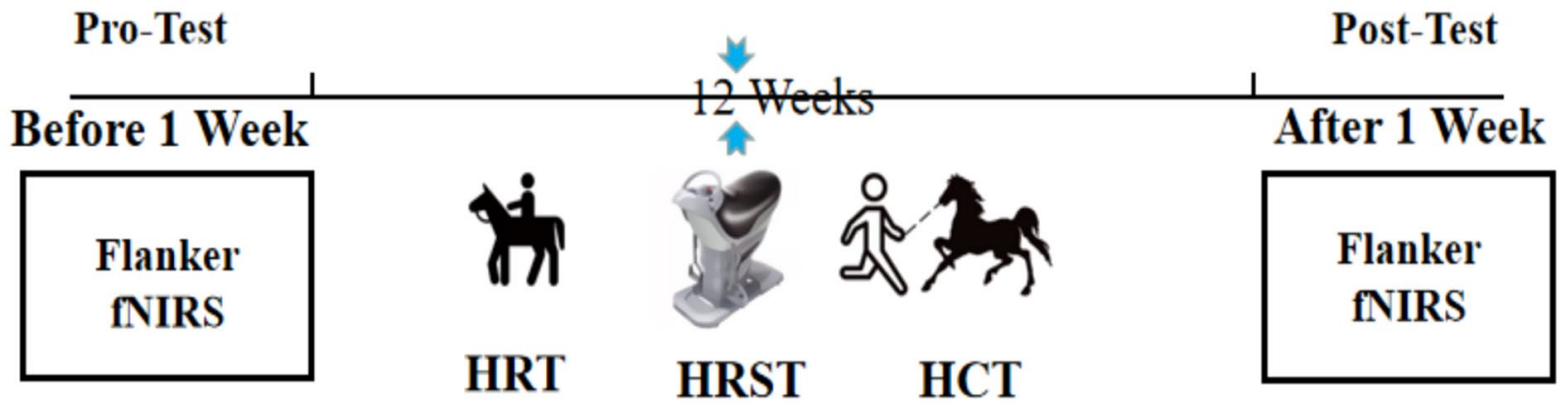
Testing and training timeline.

This experiment was an exploratory randomized controlled design. The subjects were numbered and randomly divided into three groups of 8 people using the random function in Excel 2019. Pre-test metrics, including demographic variables, Flanker task performance, and fNIRS data, were collected during the first 2 weeks of the experiment. The post-test was conducted at the end of the 12-week intervention, and the metrics were generally consistent with those from the pre-test (see Figure 1). During the experimental testing, if dizziness, nausea, vomiting, abnormal facial color, excessive fatigue, high heart rate, or other unexpected symptoms occurred, the test was immediately stopped. The experimenters were trained in relevant emergency prevention and response plans, ensuring the safety and reliability of the experiment.

### 2.2 Exercise protocol

Variations in horse size, temperament and gender can produce different intervention effects among subjects (Pluta, 2009). To ensure the safety of the experiment and the reliability of the data, given that the subjects were 7-8-year-old children, horse suitable for children’s riding were chosen. During HRT, horses move at a cadence of 60 oscillations per minute (Portaro et al., 2016). Walks are categorized as slow, fast, or running (Elmeua González and Šarabon, 2020). A slow stride is 100–120 strides per minute, which is close to the normal walking speed of adults (Mattila-Rautiainen et al., 2024). Therefore, a stride frequency of 100–120 steps per minute with 60 oscillations was selected as the training intervention requirement for the horses. In addition, to ensure the validity of the experiment, each participant rode a horse twice a week. Each session lasts approximately 40 to 50 min (Cook and Frederick, 2016). Within the 12-week HRT protocol was shown in Table 2.

**Table 2:** HRT protocol.

Time	Main content
Weeks 1–4 (basic training week)	
Week 1	Get to know each other; Instructor explains the do’s and don’ts of horseback riding; Horse food allocation under the guidance of a trainer; Feeding horses to build a trusting relationship between children and horses.
Week 2	Instructor introduces equestrian clothes and how to put them on, children put on equestrian clothes independently; Horse leading at the stable with the help of a trainer; cool down and return the horse to the stable.
Week 3	Children’s Independently Worn Equestrian Clothing; With the help of the trainer 1 person leads the horse, 1 person rides, the horse walks in a straight line at a slow trot and then alternately; cool down and return the horse to the stable.
Week 4	Warm-up, 1 person leads the horse and 1 person rides with the help of an instructor, Horses walk in a straight line at a slow trot, cool down and return the horse to the stable.
Weeks 5–8 (improvement training week)	
Week 5	Warm-up, With the help of an instructor 1 person leads the horse, 1 person rides the horse, and the horse walks in a straight line or circle at a slow pace, cool down and return the horse to the stable.
Week 6	Warm-up, With the help of the trainer 1 person leads the horse, 1 person rides the horse, the horse walks in a straight line at a fast pace and then alternates, cool down and return the horse to the stable.
Week 7	Warm-up, With the help of an instructor 1 person leads the horse, 1 person rides the horse, the horse runs in a straight line or in a circle, each person rides for 10 min and then exchanges, cool down and return the horse to the stable.
Week 8	Warm-up, With the help of the trainer 1 person leads the horse, 1 person rides the horse, and the horse performs a variable speed run, cool down and return the horse to the stable.
Weeks 9–12 (intensive training week)	
Week 9	Warm-up, With the help of the trainer 1 person leads the horse and 1 person rides the horse from a slow trot to a fast trot and joins the apparatus, take off your equestrian clothes.
Week 10	Warm-up, With the help of the trainer 1 person leads the horse, 1 person rides the horse, the horse performs a variable speed run, and equipment is added to the training process, take off your equestrian clothes.
Week 11	Warm-up, Practitioners set up obstacles, riders pass through obstacles and then switch to practice for 15–25 min per rider, take off your equestrian clothes.
Week 12	Warm-up, Practitioners set up obstacles, riders pass through obstacles and then switch to practice for 15–25 min per rider, take off your equestrian clothes.

According to the relevant literature (Cook and Frederick, 2016; Lechner et al., 2007; Künzle, 2013), the frequency generated by the vibration of the horseback riding simulator can have similarity to the impact on a person as that received by a real horse when walking. To ensure the effectiveness of the training interventions, the HRST and HCT interventions were scheduled at 40–50 min for session twice for week, for a total intervention duration of 12 weeks. The specific training protocol content of the HRST and HCT was shown in Tables 3, 4.

**Table 3:** HRST protocol.

Time	Main content
Weeks 1–4 (basic training week)	
Week 1	Adaptive horseback riding simulator
Week 2	Two hand riding simulator reins
Week 3	One hand riding simulator reins
Week 4	One hand riding simulator reins
Weeks 5–8 (improvement training week)	
Week 5	Hands side plank riding horse simulator
Week 6	Hands side plank riding horse simulator
Week 7	Reverse mount riding simulator
Week 8	Reverse mount riding simulator
Weeks 9–12 (intensive training week)	
Week 9	Backwards sitting one-handed riding horseback riding simulator
Week 10	Backwards sitting one-handed riding horseback riding simulator
Week 11	Inverted sit hands side lift riding horseback riding simulator
Week 12	Inverted sit hands side lift riding horseback riding simulator

**Table 4:** HCT protocol.

Time	Main content
Weeks 1–4 (basic training week)	
Week 1	Naming and interacting with horses
Week 2	Naming and interacting with horses
Week 3	Naming the horse, installing a water leash, stirrups, etc.
Week 4	Naming the horse, installing the bridle, stirrups, saddle, etc.
Weeks 5–8 (improvement training week)	
Week 5	Brushing, feeding, and grooming the corresponding horses
Week 6	Brushing, feeding, and grooming the corresponding horses
Week 7	Brushing, feeding, and grooming the corresponding horses
Week 8	Brushing, feeding, and grooming the corresponding horses
Weeks 9–12 (intensive training week)	
Week 9	Brushing, feeding, and grooming the corresponding horse; pulling the horse in a straight line (walk)
Week 10	Brushing, feeding, and grooming the corresponding horse; pulling the horse in a straight line (walk)
Week 11	Brushing, feeding, and grooming the corresponding horse; pulling the horse in a circle (trot)
Week 12	Brushing, feeding, and grooming the corresponding horse; pulling the horse in a circle (canter)

### 2.3 Measurement

#### 2.3.1 Flanker task

The Flanker task, a traditional conflict paradigm for studying the influence of task-irrelevant information on processing task-relevant information (Ulrich et al., 2021), was used for assessing inhibitory control (Diamond, 2013). The Flanker task written in E-prime 2.0 (Psychology Software Tools Inc., Pittsburgh, PA, USA), and the test metrics included accuracy and reaction time (RT) in the Flanker congruent and incongruent task situations. During the experimental task, the participants were instructed to focus on the “+” symbol in the center of the screen as a cue to start the task. Subsequently, a sequence of five letter combinations was displayed on the screen for 1,000 ms, with the middle arrow serving as the point of gaze and a stimulus interval of 1 s. This sequence of letters can occur in the following two conditions: consistent conditions, such as “FFFFF” and “LLLLL,” and inconsistent conditions, such as “LLFLL” and “FFLFF.” The participants were required to respond as quickly and correctly as possible to the middle letter of each sequence by pressing the “F” key on the keyboard with their index finger if it was an “F” and the “L” key if it was an “L.” The two conditions were presented in an equal and randomized manner, with the formal test comprising two segments, each of which required 60 judgments and 12 practice sessions before the formal test. Only use the correct experiments to analyze the RT data. Incorrect experiments were included in the accuracy analysis but were excluded from the RT analysis. Before calculating the average RT of each participant under each task condition, extremely fast or extremely slow reactions and obvious outliers were examined.

#### 2.3.2 fNIRS measurements

**Figure 2:**
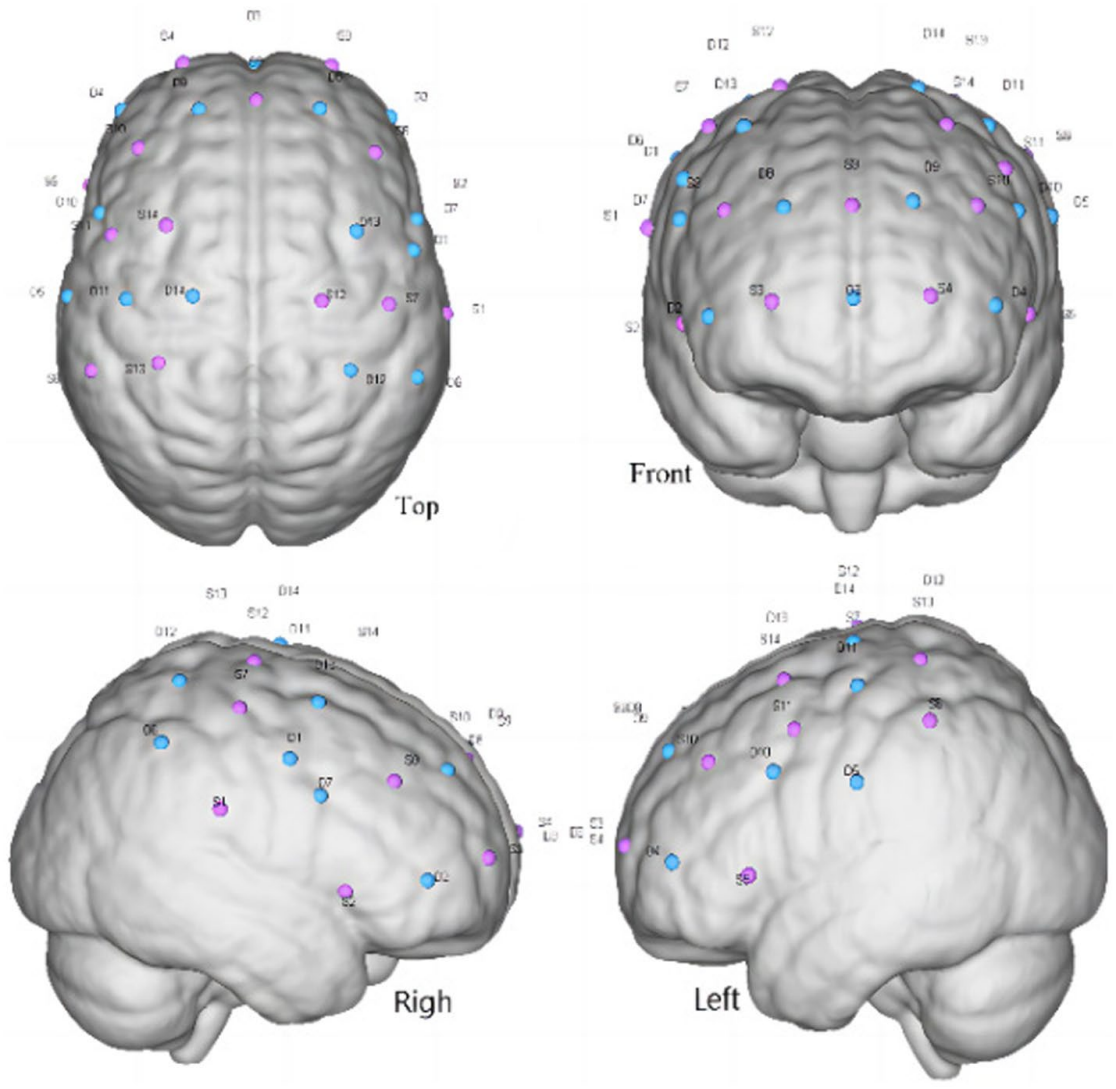
Signal sources and detectors in the prefrontal and motor cortex (Cheng et al., 2023).

A portable functional near-infrared spectrometer (NirSmart63) captured the raw signal light intensity in the prefrontal and motor areas, and then converted the captured data into mean Oxy-Hb change values via the NirSpark 1.7.5 software package. Related studies indicate that Oxy-Hb data are commonly used to assess brain activation during cognitive tasks (Sato et al., 2004). The raw light intensity signal was visually inspected and processed in NirSpark. 1.7.5. Use the software’s motion artifact handler to correct motion-contaminated fragments and/or exclude artifacts if they cannot be adequately corrected. The signal is then bandpass filtered to attenuate slow drift and high-frequency physiological noise. The changes in Oxy-Hb and Deoxy-Hb were calculated from the optical density using a modified Beer–Lambert law. For task-based analyses, baseline was defined as the pre-stimulation period (fixed) before each trial, and hemoglobin changes were quantified relative to this baseline. Channels with poor optometry-scalp coupling, signal saturation, or excessive noise (based on instrument quality indicators and visual inspection) were marked as noisy and excluded from subsequent analysis. These data were tested as follows. Changes in cerebral blood oxygen concentration in the prefrontal and motor regions during the Flanker task were monitored using a portable NirSmart63. This device utilized three near-infrared wavelengths (780 nm, 805 nm, 830 nm) to record raw signal light intensity, with a sampling frequency set to 11 Hz (Cheng et al., 2023). The acquisition system used in this experiment had 14 sources and 14 detectors. The prefrontal and motor areas were positioned according to the Brodmann partitions (as shown in Figure 2, illustrating the upper, anterior, right, and left loci), with the probes arranged in a regular pattern.

### 2.4 Statistics and analysis

Experimental results are presented as mean ± standard deviation (M ± SD). Variability was assessed using one-way ANOVA, and normality was tested with the Shapiro–Wilk test. Flanker test results were analyzed using a 3 (group: HRTG, HRSG, and HCG) × 2 (time: pre and post-test) repeated measures ANOVA. If a significant group × time interaction was found, a simple effects analysis was performed. Differences in brain activation between pre and post-tests were assessed using paired samples *t*-tests. All statistical analyses were performed using SPSS26.0, with *p* < 0.05 considered statistically significant. Results with 0.05 ≤ *p* < 0.10 are described as trend-level (marginal) effects. In the tables, statistically significant results are highlighted in bold, and trend-level results (if any) are indicated with a dagger (*) symbol. Given the exploratory nature of this study and the number of behavioral endpoints and channel fNIRS comparisons, we note that the reported channel fNIRS *p*-values were not adjusted for multiple comparisons and should be interpreted with caution as hypothesis generation rather than validation. Where feasible, we report the effect size of the combined effect (e.g., partial *η*^2^ and the normalized mean difference of the key contrasts) to help explain the size and practical relevance of the findings. Future studies with sufficient motivation will pre-specify multiplicity control procedures (e.g., false discovery rates) for confirmatory inference and will systematically report confidence intervals.

Flanker test results were analyzed using a 3 (group: HRTG, HRSG, and HCG) × 2 (time: pre-test and post-test) repeated-measures ANOVA. If a significant group × time interaction was found, simple effects analysis was performed. Differences in brain activation between pre- and post-tests were assessed using paired-samples *t*-tests. All statistical analyses were performed using SPSS 26.0, with *p* < 0.05 considered statistically significant. Results with 0.05 ≤ *p* < 0.10 were described as trend-level marginal effects. In the tables, statistically significant results were highlighted in bold, and trend-level results, if any, were indicated with a dagger (*) symbol. Given the exploratory nature of this study and the number of behavioral endpoints and channel fNIRS comparisons, the reported channel fNIRS *p*-values were not adjusted for multiple comparisons and should be interpreted with caution as hypothesis-generating rather than confirmatory. Where feasible, the effect size of the combined effect, such as partial *η*^2^ and the normalized mean difference of the key contrasts, was reported to help explain the size and practical relevance of the findings. Future studies with sufficient power should pre-specify multiplicity control procedures, such as false discovery rate correction, for confirmatory inference and systematically report confidence intervals.

## 3 Results

### 3.1 Demographic variables

As shown in Table 5, the demographic variables include age, height, weight and BMI. The data were analyzed using one-way ANOVA on the samples of the three groups pre-test as a way to reduce the impact on the experimental results.

**Table 5:** Demographic data difference comparison.

Measures	HRTG (*N* = 8)	HRSG (*N* = 8)	HCG (*N* = 8)	*F*	*p*
Years	7.5	7.5	7.5	–	–
Height (cm)	128.50 ± 7.35	128.13 ± 5.82	127.38 ± 5.10	0.01	0.94
Weight (kg)	27.63 ± 2.82	26.96 ± 3.53	27.16 ± 2.27	0.11	0.89
BMI (kg/m^2^)	16.73 ± 1.16	16.36 ± 1.08	16.72 ± 0.61	0.37	0.70

All data were normally distributed (*p* > 0.05) and variance aligned (*p* > 0.05) before one-way ANOVA was performed. The height (cm) of the three groups were HRTG: 128.50 ± 7.35, HRSG: 128.13 ± 5.82 and HCG: 127.38 ± 5.10, *F* = 0.01, *p* = 0.94 > 0.05; the weight (kg) of the three groups were HRTG: 27.63 ± 2.82, HRSG: 26.96 ± 3.53 and HCG: 27.16 ± 2.27, *F* = 0.11, *p* = 0.89 > 0.05; BMI (kg/m^2^) of the three groups were HRTG: 16.73 ± 1.16, HRSG: 16.36 ± 1.08 and HCG: 16.72 ± 0.61, *F* = 0.37, *p* = 0.70 > 0.05; the above differences were not statistically significant at *p* > 0.05. Therefore, there were no significant differences in demographic variables among the three experimental groups.

### 3.2 Behavioral data variation

To investigate the effects of three different training interventions on the IC of children age 7–8 years, a 3 (group: HRTG, HRSG, and HCG) × 2 (time: pre-test, post-test) repeated-measures ANOVA was conducted on the data from the three task tests pre and post-test. The statistical results are presented in Table 6.

**Table 6:** Variance statistics of Flanker scores pro and post three different training.

Effect	*df*	*F*	*p*	*η*²_partial_
Congruent task accuracy (%)				
Time	1	4.56	**0 0.04** ^*^	0.18
Time × group	2	3.95	**0 0.03**	0.27
Error	21			
Group	2	0.12	0.77	0.01
Error	21			
Incongruent task accuracy (%)				
Time	1	7.16	**0 0.02** ^*^	0.25
Time × group	2	1.05	0.37	0.01
Error	21			
Group	2	0.34	0.62	0.01
Error	21			
Congruent task RTs (ms)				
Time	1	4.05	0.06	0.16
Time × group	2	4.83	**0.02** ^*^	0.32
Error	21			
Group	2	1.70	0.21	0.14
Error	21			
Incongruent task RTs (ms)				
Time	1	7.71	**0.013** ^*^	0.27
Time × group	2	4.02	**0.03** ^*^	0.28
Error	21			
Group	2	0.39	0.58	0.04
Error	21			

RTs, reaction time, ^*^p < 0.05, ^**^p < 0.01.

In the congruent task accuracy score variable, there was no significant main effect of group, *F*_(2,21)_ = 0.12, *p* = 0.77, *η*^2^_partial_ = 0.01, and a significant main effect of time, *F*_(1,21)_ = 4.56, *p* = 0.04, *η*^2^_partial_ = 0.18, and there was also a significant interaction effect of group × time, *F*_(2,21)_ = 3.95, *p* = 0.03, *η*^2^_partial_ = 0.27 (see Table 6). A simple effects analysis showed that HRTG was significantly higher after the intervention (*p* < 0.01), pro and post the intervention: 0.84 ± 0.07 and 0.89 ± 0.06, respectively (see Table 7).

**Table 7:** Comparison of differences in Flanker scores pro and post-test three different training interventions.

Task condition	Group	Pro	Post	Changes intergroups
				Mean value difference
Congruent task accuracy (%)	HRTG	0.84 ± 0.07	0.89 ± 0.06	**0.05** ^**^
	HRSG	0.85 ± 0.07	0.86 ± 0.06	0.01
	HCG	0.85 ± 0.07	0.84 ± 0.07	−0.01
	Discrepancy	−0.01/−0.01/−0.01	0.03/0.05/0.02	
Incongruent task accuracy (%)	HRTG	0.82 ± 0.07	0.85 ± 0.05	**0.03** ^**^
	HRSG	0.85 ± 0.07	0.85 ± 0.05	0.01
	HCG	0.84 ± 0.09	0.85 ± 0.07	0.03
	Discrepancy	−0.03/−0.02/−0.01	0.01/−0.02/−0.02	
Congruent task RT (ms)	HRTG	695.41 ± 96.68	634.41 ± 63.29	**−61.00** ^**^
	HRSG	687.37 ± 80.15	679.42 ± 78.25	−7.95
	HCG	724.27 ± 65.03	727.95 ± 65.98	3.68
	Discrepancy	8.04/−28.86/−36.9	−45.01/−95.54/−48.53	
Incongruent task RT (ms)	HRTG	758.20 ± 100.44	691.21 ± 73.80	**−66.99** ^**^
	HRSG	755.89 ± 134.27	742.84 ± 125.75	−13.05
	HCG	764.03 ± 73.15	757.73 ± 84.23	−6.3
	Discrepancy	2.31/−5.83/−8.14	−51.63/−66.52/−14.89	

RTs = reaction time, ^*^p < 0.05, ^**^p < 0.01, Discrepancy = difference between groups.

In the incongruent task accuracy score variable, there was no significant main effect of group, *F*_(2,21)_ = 0.34, *p* = 0.62, *η*^2^_partial_ = 0.01, and a significant main effect of time, *F*_(1,21)_ = 7.16, *p* = 0.02, *η*^2^_partial_ = 0.25, but there was no significant interaction effect of group × time, *F*_(2,21)_ = 1.05, *p* = 0.37, *η*^2^_partial_ = 0.01 (see Table 6). After passing the 12-week intervention: the HRTG was significantly higher after the intervention (*p* < 0.01), pro and post the intervention: 0.82 ± 0.07 and 0.85 ± 0.05, respectively (see Table 7).

In the congruent task RTs (ms) score variable, there were no significant main effects of group, *F*_(2,21)_ = 1.70, *p* = 0.21, *η*^2^_partial_ = 0.14, and a trend-level main effect of time, *F*_(1,21)_ = 4.05, *p* = 0.06, *η*^2^_partial_ = 0.16, but there was a significant interaction effect of group × time, *F*_(2,21)_ = 4.83, *p* = 0.02, *η*^2^_partial_ = 0.32 (see Table 6). A simple effects analysis showed that the HRTG was significantly reduced after the intervention (*p* < 0.01), before and after the intervention: 695.41 ± 96.68 and 634.41 ± 63.29, respectively (see Table 7).

In the Incongruent task RTs (ms) score variable, there was no significant main effect of group, *F*_(2,21)_ = 0.39, *p* = 0.58, *η*^2^_partial_ = 0.04, but there was a significant main effect of time, *F*_(2,21)_ = 7.71, *p* = 0.013, *η*^2^_partial_ = 0.27, and there was also a significant interaction effect of group × time, *F*_(2,21)_ = 4.02, *p* = 0.03, *η*^2^_partial_ = 0.28 (see Table 6). A simple effects analysis showed that HRTG was significantly reduced after the intervention (*p* < 0.01), before and after the intervention: 758.20 ± 100.44 and 691.21 ± 73.80, respectively (see Table 7).

### 3.3 fNIRS results

#### 3.3.1 Activation of brain regions

**Figure 3:**
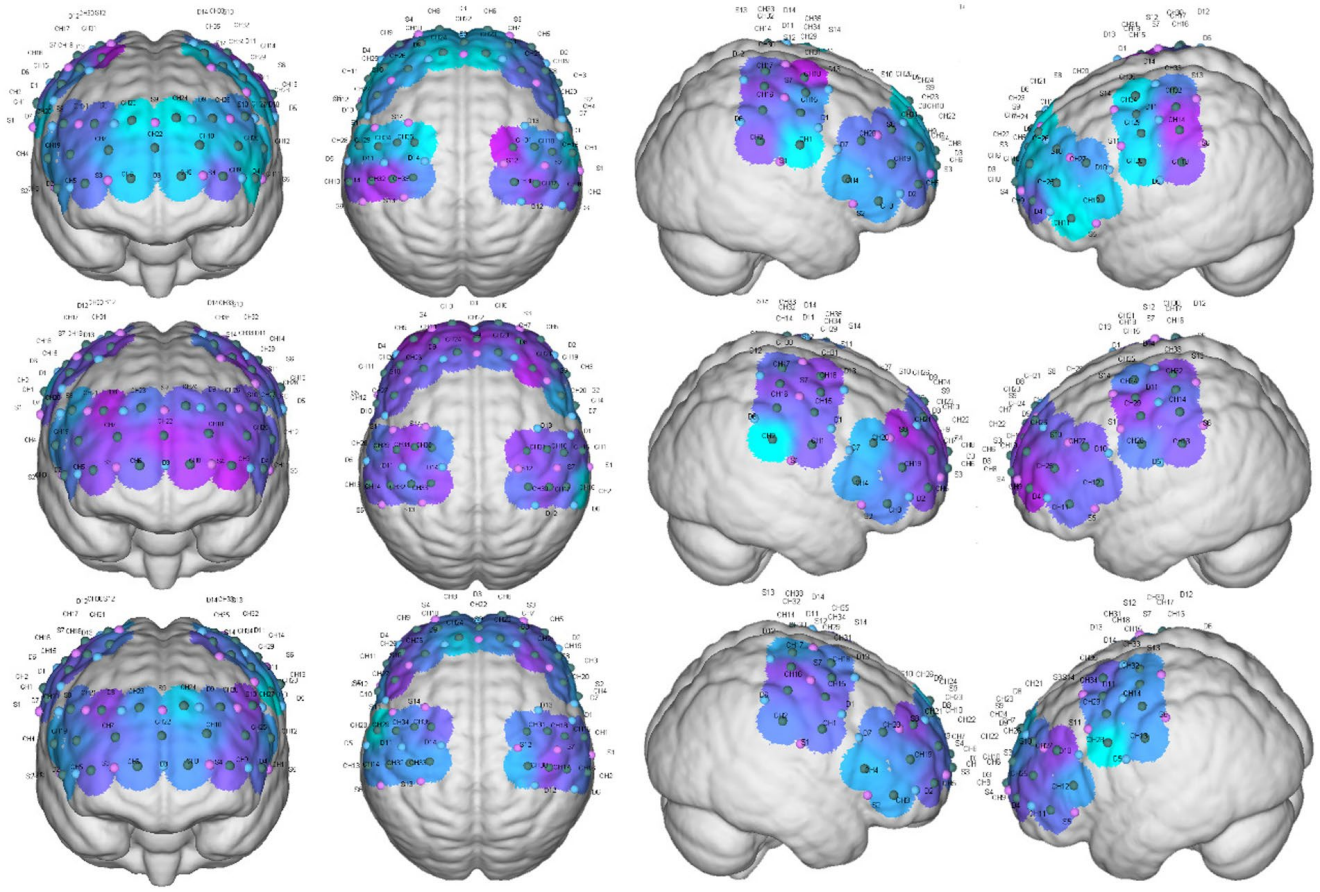
Activation maps of prefrontal and motor areas pro-test in the Flanker congruency task in three groups (each row was: HRTG/HRSG/HCG, respectively).

**Figure 4:**
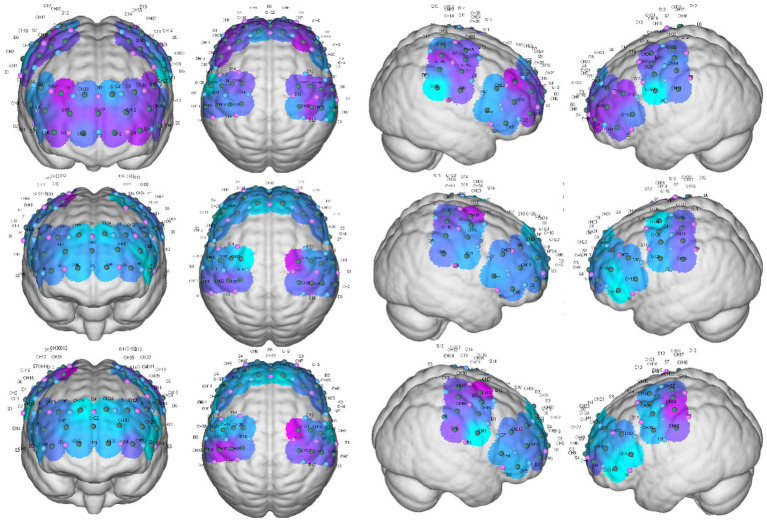
Activation maps of prefrontal and motor areas post-test in the Flanker congruency task in three groups (each row was: HRTG, HRSG, HCG, respectively).

**Figure 5:**
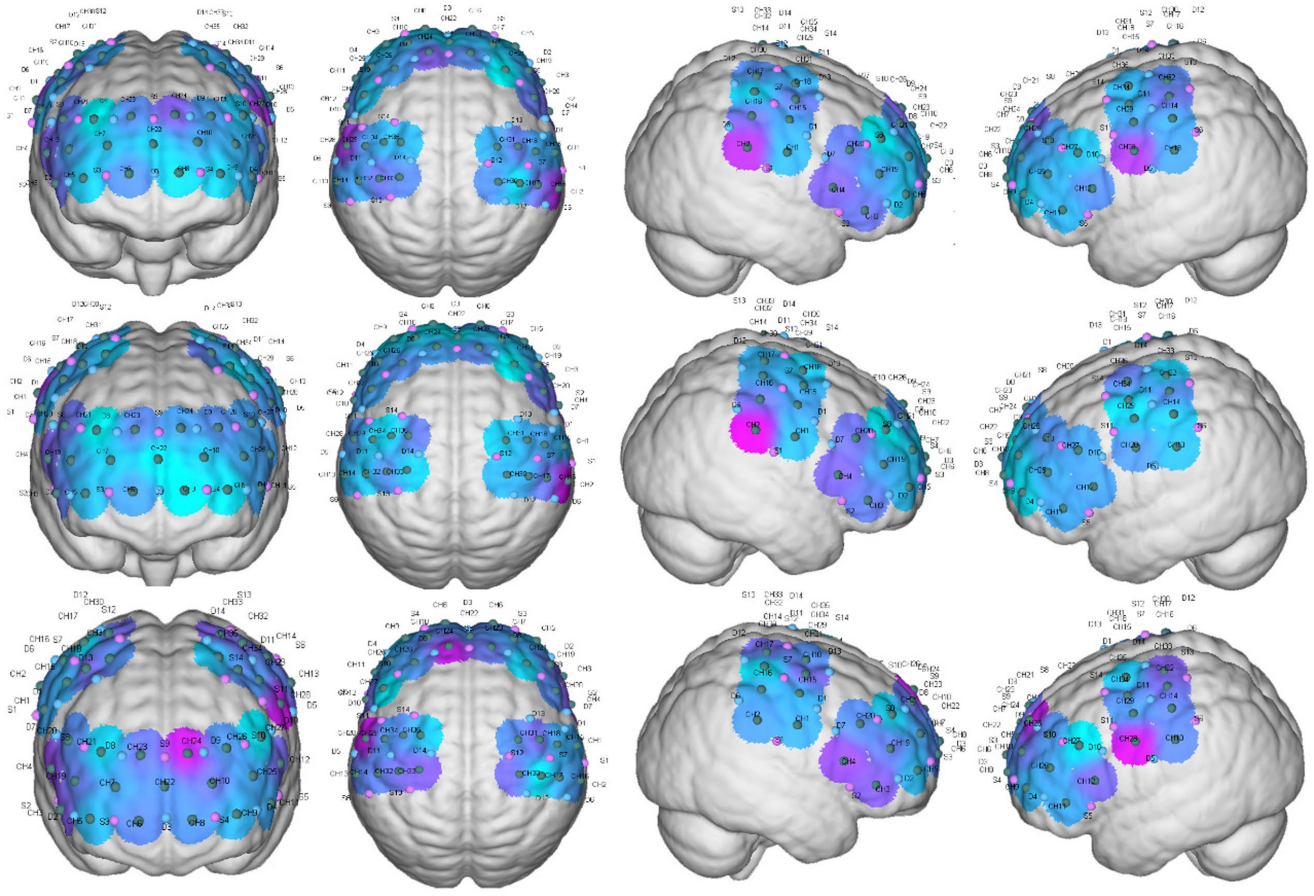
Activation maps of prefrontal and motor areas pro-test in the Flanker incongruent task in three groups (each row was: HRTG/HRSG/HCG, respectively).

**Figure 6:**
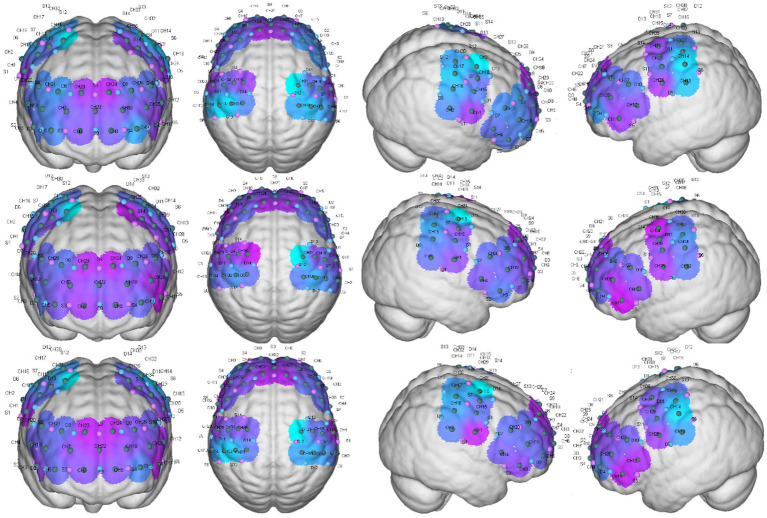
Activation maps of prefrontal and motor areas post-test in the Flanker incongruent task in three groups (each row was: HRTG/HRSG/HCG respectively).

In short, Figures 3–6 provide a qualitative visualization of the task-induced Oxy-Hb changes in each group of prefrontal and motor regions; Tables 8, 9 report the quantitative channel statistics.

**Table 8:** Table of mean channel changes in Oxy-Hb concentration in prefrontal and motor areas pro and post-test the flanker congruency task.

Channel	S-D	HRTG		T	P	HRSG		T	P	HCG		T	P
		Pro	Post			Pro	Post			Pro	Post		
1	S1-D1	−0.003	0.004	1.34	0.27	0.003	0.000	0.67	0.57	0.006	−0.005	1.29	0.33
2	S1-D6	0.006	−0.004	−0.78	0.49	−0.005	0.003	−0.44	0.70	0.005	0.011	−0.42	0.72
3	S2-D2	0.001	0.001	0.08	0.94	0.001	0.001	0.06	0.95	0.001	0.000	0.21	0.85
4	S2-D7	0.001	0.000	−0.49	0.65	0.000	−0.001	0.09	0.94	0.000	0.002	−1.01	0.42
5	S3-D2	0.004	0.006	0.39	0.71	0.004	0.001	0.57	0.61	0.008	0.006	0.38	0.74
6	S3-D3	−0.001	0.003	0.89	0.42	0.003	−0.002	0.93	0.42	0.005	0.002	0.86	0.48
7	S3-D8	−0.001	0.005	4.29	**0.013** ^*^	0.005	−0.002	1.28	0.29	0.007	0.001	0.95	0.44
8	S4-D3	0.000	0.006	1.28	0.27	0.005	−0.001	3.19	0.05	0.004	0.001	1.24	0.34
9	S4-D4	0.005	0.007	0.29	0.79	0.007	0.001	0.74	0.51	0.009	0.008	0.09	0.94
10	S4-D9	0.004	0.008	0.84	0.45	0.005	−0.001	2.50	0.09	0.003	−0.002	0.88	0.47
11	S5-D4	−0.004	0.004	1.91	0.13	0.003	−0.007	1.78	0.17	0.006	−0.006	1.54	0.26
12	S5-D10	−0.002	0.002	1.87	0.14	0.003	−0.001	1.42	0.25	0.002	−0.004	1.84	0.21
13	S6-D5	0.007	0.003	−0.94	0.42	0.003	0.004	−0.17	0.88	0.003	0.010	−0.77	0.52
14	S6-D11	0.009	0.003	−0.87	0.45	0.003	0.005	−0.22	0.85	0.003	0.016	−1.11	0.38
15	S7-D1	0.003	0.005	0.41	0.71	0.003	0.002	0.22	0.85	0.009	0.006	0.26	0.82
16	S7-D6	0.005	0.002	0.02	0.99	0.002	0.002	0.40	0.73	0.008	0.008	−0.01	1.00
17	S7-D12	0.008	0.008	0.04	0.97	0.005	0.007	−0.35	0.76	0.012	0.013	−0.10	0.93
18	S7-D13	0.001	0.005	0.77	0.50	0.004	−0.002	0.83	0.49	0.007	0.004	0.35	0.76
19	S8-D2	0.004	0.003	−0.07	0.95	0.003	0.001	0.45	0.68	0.003	0.005	−0.31	0.79
20	S8-D7	0.004	0.000	−0.67	0.54	−0.001	0.002	−0.40	0.72	0.005	0.006	−0.11	0.92
21	S8-D8	−0.002	0.004	3.08	**0.041** ^*^	0.007	0.002	0.67	0.55	0.011	0.005	0.90	0.46
22	S9-D3	−0.004	0.002	2.05	0.11	0.006	0.000	3.12	0.05	0.004	0.000	1.39	0.30
23	S9-D8	0.000	0.006	4.09	**0.016** ^*^	0.002	−0.004	1.53	0.22	0.003	−0.006	1.94	0.28
24	S9-D9	−0.004	0.000	0.89	0.42	0.003	−0.005	2.44	0.17	−0.003	−0.003	−0.10	0.93
25	S10-D4	−0.002	0.006	1.33	0.25	0.004	−0.005	1.22	0.31	0.009	0.001	0.75	0.53
26	S10-D9	0.000	0.004	1.16	0.31	0.003	0.000	0.81	0.48	0.005	0.000	0.74	0.53
27	S10-D10	0.002	0.006	0.80	0.47	0.003	0.000	0.48	0.67	0.012	0.004	1.07	0.40
28	S11-D5	−0.002	−0.005	−0.28	0.79	0.002	−0.001	0.59	0.62	−0.007	0.001	−0.58	0.62
29	S11-D11	−0.002	0.002	0.79	0.49	0.005	0.000	0.73	0.54	0.003	−0.002	0.38	0.74
30	S12-D12	0.005	0.001	−0.80	0.48	0.002	0.004	−0.19	0.87	0.000	0.009	−1.11	0.38
31	S12-D13	0.011	0.004	−0.85	0.46	0.005	0.011	−0.67	0.57	0.004	0.019	-1.72	0.23
32	S13-D11	0.009	0.001	−1.02	0.38	0.002	0.006	−0.47	0.68	0.002	0.015	-0.95	0.44
33	S13-D14	0.003	0.003	−0.02	0.99	0.004	0.004	0.06	0.96	0.002	0.006	-0.32	0.78
34	S14-D11	0.000	0.005	1.00	0.39	0.006	−0.002	2.27	0.15	0.004	0.002	0.25	0.83
35	S14-D14	−0.003	0.004	0.97	0.40	0.001	−0.006	0.72	0.54	0.008	0.003	0.72	0.55

Statistically significant results (*p* < 0.05) are highlighted in bold font. Trend-level results (0.05 ≤ *p* < 0.10), if present, are marked with *.

**Table 9:** Table of mean channel changes in Oxy-Hb concentration in prefrontal and motor areas pro and post-test the Flanker incongruency task.

Channel	S-D	HRTG		*T*	*p*	HRSG		*T*	*p*	HCG		*T*	*p*
		Pro	Post			Pro	Post			Pro	Post		
1	S1-D1	0.0141	−0.0176	2.05	0.13	−0.0032	0.0039	1.28	0.29	0.0008	0.0002	0.18	0.87
2	S1-D6	0.0069	−0.0004	0.48	0.66	0.0045	−0.004	−0.51	0.65	0.0032	0.0035	−0.2	0.85
3	S2-D2	0.0263	0.0135	0.58	0.60	0.0001	−0.002	−1.37	0.24	−0.0054	−0.006	0.26	0.81
4	S2-D7	0.0364	0.0023	2.22	0.09	0.0013	−0.002	−1.38	0.240	−0.0032	−0.002	0.04	0.97
5	S3-D2	−0.004	0.0178	−1.33	0.25	−0.0049	−0.003	0.43	0.69	−0.0027	−0.002	0.88	0.44
6	S3-D3	0.0063	0.0070	−0.21	0.84	−0.0013	0.0007	0.47	0.67	−0.0015	−0.002	−0.59	0.6
7	S3-D8	0.0024	0.0065	−0.62	0.57	−0.0044	−0.001	1.51	0.21	−0.0056	−0.004	0.93	0.42
8	S4-D3	0.0000	0.0106	−0.78	0.48	−0.0053	0.0000	2.59	0.06	−0.0034	−0.007	−1.37	0.26
9	S4-D4	0.0177	0.0054	0.57	0.60	−0.0052	−0.00	0.02	0.99	−0.0029	−0.002	2.7	0.07
10	S4-D9	−0.005	−0.0011	−0.25	0.82	−0.0029	0.0006	1.37	0.24	0.0012	0.0018	0.35	0.75
11	S5-D4	0.0080	0.0162	−0.66	0.54	−0.0036	0.0030	1.51	0.21	−0.0018	−0.003	−1.45	0.24
12	S5-D10	0.0145	0.0038	0.67	0.54	−0.0012	0.0013	1.40	0.24	−0.0002	−0.001	−1.32	0.28
13	S6-D5	−0.013	0.0062	−0.80	0.48	−0.0022	−0.008	−0.75	0.51	−0.0044	−0.004	−0.77	0.5
14	S6-D11	−0.001	0.0177	−0.94	0.42	−0.0026	−0.011	−0.69	0.54	0.0044	0.0034	−0.11	0.92
15	S7-D1	0.0198	0.0039	0.36	0.74	−0.0028	−0.004	0.08	0.94	0.0034	0.0047	1.03	0.38
16	S7-D6	−0.001	0.0198	−2.16	0.12	0.0007	−0.007	−0.33	0.76	0.0063	0.0075	0.42	0.7
17	S7-D12	−0.000	0.0331	−0.94	0.42	0.0067	−0.009	−0.08	0.94	−0.0011	−0.002	0.49	0.66
18	S7-D13	0.0017	0.0329	−0.60	0.59	0.0002	−0.001	0.00	1.00	0.0065	0.0064	−0.67	0.55
19	S8-D2	−0.004	0.0203	−1.68	0.17	0.0024	−0.003	−0.25	0.81	−0.0034	0.0028	2.88	0.06
20	S8-D7	0.006	0.0141	−0.18	0.87	−0.0003	−0.003	−0.61	0.57	−0.0029	−0.001	0.99	0.39
21	S8-D8	−0.012	0.0071	−1.28	0.27	−0.0075	−0.004	0.77	0.48	−0.006	−0.007	−0.81	0.48
22	S9-D3	0.0143	0.0028	0.56	0.61	−0.0052	0.0013	2.71	0.06	0.0049	0.0021	−1.29	0.29
23	S9-D8	0.0029	0.0042	−0.17	0.87	−0.0012	0.0036	1.64	0.18	0.0058	0.0039	−0.48	0.67
24	S9-D9	−0.016	0.0248	−1.91	0.13	0.0017	0.0048	0.88	0.43	−0.0032	−0.005	−0.54	0.62
25	S10-D4	0.013	0.0083	0.44	0.68	−0.0034	0.0014	0.83	0.45	−0.0045	−0.002	2.01	0.14
26	S10-D9	−0.013	0.0122	−1.55	0.20	−0.0024	−0.001	0.17	0.87	−0.0052	−0.005	−0.87	0.45
27	S10-D10	−0.006	0.0317	−2.09	0.10	−0.0046	−0.002	0.47	0.66	0.0067	0.007	−0.14	0.89
28	S11-D5	−0.001	0.0141	−2.89	0.06	0.0038	0.0038	−0.04	0.97	0.0012	−0.001	−0.59	0.59
29	S11-D11	−0.012	0.0143	−1.24	0.30	−0.0021	−0.002	0.17	0.88	−0.0067	−0.005	0.39	0.72
30	S12-D12	−0.001	0.0173	−1.80	0.17	−0.0013	−0.006	−0.57	0.61	0.0065	0.0061	−1.11	0.35
31	S12-D13	−0.007	0.0020	−0.32	0.77	−0.0033	−0.015	−0.83	0.47	−0.0002	−0.002	−0.5	0.65
32	S13-D11	−0.002	0.0406	−2.72	0.07	0.0006	−0.011	−1.07	0.36	0.007	0.0083	1.5	0.23
33	S13-D14	0.008	0.0179	−0.39	0.72	−0.0011	−0.003	−0.16	0.88	0.0033	0.0024	−0.76	0.5
34	S14-D11	−0.004	0.0209	−0.70	0.53	−0.0037	0.0017	1.34	0.27	0.0029	0.0015	−1.32	0.28
35	S14-D14	0.0007	0.0131	−0.57	0.61	−0.0036	0.0031	0.86	0.45	−0.0021	−0.004	−1.55	0.22

Trend-level results (0.05 ≤ *p* < 0.10), if present, are marked with *.

During the test, each pair of neighboring light sources and detectors formed a channel. The values of each channel were averaged and then processed using the inverse distance interpolation method to generate an image (Song et al., 2022). This image represents the activation map of the mean change in Oxy-Hb for each channel in the prefrontal and motor area pre and post the experiment. These maps provide a qualitative, channel-level topographic visualization of mean Oxy-Hb changes during the Flanker task. Given the spatial resolution of fNIRS and the opcode arrangement, the maps should be interpreted as approximate and do not imply precise cortical localization, as shown in Figures 3–6.

#### 3.3.2 Differences in brain area activation

Under the Flanker congruent task, exploratory, unadjusted channel-wise analyses suggested increased Oxy-Hb in HRTG within channels approximately over the right lateral prefrontal cortex (putative R-DLPFC; Ch7/21/23), whereas no significant pre–post changes were observed in HRSG and HCG.

All three groups conducted pre-and post-tests for the Flanker task, with raw signals collected using NirSmart63. The raw signals were processed using NirSpark 1.7.5 to calculate changes in Oxy-Hb in each channel within the prefrontal and motor regions for each group. The values for each channel in the 24 subjects were averaged, and a paired-sample *t*-test was conducted to determine the differences in mean Oxy-Hb changes before and after the tests in the three groups. The specific results are presented in Tables 8, 9.

Given the exploratory nature of the channel-wise analyses, the main focuses on ROI-level interpretation (prefrontal/DLPFC-related findings), with full channel-wise outputs provided in Tables 8, 9. The fNIRS results, divided by channels, were reported as exploratory observations (unadjusted *p*-values). Under the Flanker congruent condition, the HRT group showed increased Oxy-Hb within the channels comprising the *a priori* right lateral prefrontal (putative R-DLPFC) ROI (Ch7/21/23), while no significant pre–post changes were observed in HRSG and HCG. Under the Flanker incongruency task, no significant anterior–posterior channel changes were detected in any group. Tables 8, 9 provide the complete channel output. These channel-level observations are intended to characterize the topography of hemodynamic changes induced by the task and should not be interpreted as evidence of precise cortical localization or confirmed neural mechanisms.

## 4 Discussion

After 12 weeks, we observed preliminary, task-specific improvements in Flanker performance in the HRTG. In parallel, exploratory fNIRS findings suggested increased task-evoked Oxy-Hb during the congruent task within channels comprising an a priori right lateral prefrontal (putative R-DLPFC) ROI (Ch7/21/23). Given the small sample size and unadjusted channel-wise testing, these findings should be interpreted as hypothesis-generating rather than confirmatory evidence of a specific neural mechanism. Thus, the findings of fNIRS should be regarded as exploratory neural evidence consistent with behavioral improvements, rather than direct statistical evidence that neural activation mediates behavioral changes.

IC is the ability to manage attention, behavior, thoughts and emotions to overcome strong internal biases or distraction from external stimuli and to respond best in a given situation (Amatriain-Fernández et al., 2021). Relevant studies have shown that the sensitive period for the development of IC was stage-specific, with 6–7 years of age being the sensitive period for development, slow growth after 7 years of age, and leveling off after 10 years of age. The subjects in this study were children aged 7–8 years, who were in the sensitive period of inhibition development when training intervention are beneficial for the rapid growth of IC. In addition, findings in other exercise programs are consistent with the present study (Ishihara et al., 2017; Su-Youn et al., 2017; Ishihara and Mizuno, 2018), suggesting that exercise at certain loads can be effective in improving inhibition. Ishihara et al. (2017) found that children aged 6–15 years old who regularly participated in tennis matches showed better inhibition. Su-Youn et al. (2017) studied the inhibition of 30 Taekwondo elementary school students using the Stroop test to assess their IC. The results of this study showed that the levels of neuroplasticity-related growth factors could be induced by training increase as a way to improve children inhibitory. Moreover, it has also been shown that a 12–24 weeks chronic exercise cycle with 50–90 min/week of exercise and a frequency of 2–3 times/week significantly affects IC (Li, 2021).

In fact, both simple and complex technical movements are regulated and controlled by the nervous system, with the brain playing the most significant and prominent role in regulation (Cheng et al., 2023). Relevant studies have found that sports with higher cognitive involvement are effective in improving children’s cognitive abilities compared to sports with lower cognitive involvement (Best and Miller, 2010), which also provides evidence for the increase in inhibition in the present study. Riders must perform multiple inhibitions when faced with changing signals and postures in different environments. For example, when children aged 7–8 years perform a double arm lateral raise they are exposed to the shock of the sine wave generated by the horse’s walk and the interference of the plane of instability in the three-axis movement pattern, which forces the rider to integrate their own tactile and proprioceptive senses, coordinate the upper and lower limbs to generate force, and pass the adductor muscles on the medial side of the thighs, suture muscles and medial femoral muscles to coordinate the force to give the horse cues and reduce the walking speed to maintain body balance. Through this positive feedback and transmission to the brain, the rider may unconsciously develop positive inhibitions on the body, further improving the rider’s cognitive abilities (Cheng et al., 2023). Other studies have also shown that HRT is effective in improving children’s attention and social engagement (Ward et al., 2013), further improving inhibition and social interaction.

Relevant studies have shown that the prefrontal cortex has extensive connections to a wide variety cortical and subcortical structures and is closely linked to attention, interference control, response inhibition, and the selection, maintenance, and manipulation of task-relevant information in working memory (Banich et al., 2000). These indicators of cognitive control continue to improve throughout childhood, with the frontal cortex playing an important role in changes in extrinsic behavioral performance (Chaddock-Heyman et al., 2014), which is associated with cortical activation during the performance of various cognitive tasks in the children aged 7–8 years in this study.

The fact that increased Oxy-Hb levels reflect increased local cerebral blood flow triggered by the activation of neuronal cells in the cerebral cortex suggests more intense activation in the corresponding brain regions of the brain. According to the principle of neuronal cell coupling, an increase in local oxygen supply to the brain indicates that the brain needs to recruit more neuronal resources (Suzuki et al., 2004) and that the cortical activation of the brain is enhanced (Byun et al., 2014).

It has been shown that high rates of correctness during the performance of attentional network tasks may be associated with high neural activation of the right frontal–parietal network (Xuan et al., 2016) and show a clear right hemisphere dominance (Thiebaut de Schotten et al., 2011). Prior work has implicated lateral prefrontal regions, including the right DLPFC, in executive-control demands during Flanker-type tasks. This literature informed our *a priori* focus on a right lateral prefrontal ROI covered by our optode montage. However, given the exploratory and uncorrected nature of the present channel-level analyses, the current findings do not establish anatomical specificity and require replication in larger samples with pre-specified ROI and multiplicity procedures. It has been shown that the brain activation sites associated with the Flanker task are mainly found over channels approximately over the right lateral prefrontal cortex (putative R-DLPFC region) (Nee et al., 2007) and that different types of cue conditions are associated with frontal–parietal network activation, which is in line with our results, through the counting of several data closely related to the activation modeling of brain regions in the Flanker task. Whereas the R-DLPFC is considered an important component of the right front-parietal network (Wang et al., 2016), greater recruitment of this network has been associated with better performance on executive-control tasks.

The same results were found in other sports. Yu et al. (2023) found that all three groups significantly activated the right dorsolateral prefrontal cortex by comparing between elite, expert and novice athletes, and Cheng et al. (2023) investigated the changes and brain neural mechanisms of EAA on inhibitory ability of children aged 7–8 years by 12 weeks of HRT intervention. The results showed that HRT may be associated with improvements inhibitory ability and increased task-evoked activation in channels approximately over the right dorsolateral prefrontal cortex in children aged 7–8 years. In addition, it has also been shown that physical exercise can promote the secretion of substances such as catecholamine in the prefrontal cortex of the human body, increase the blood flow in the cerebral cortex, and optimize the allocation of cognitive resources, which is to some extent conducive to the optimization of cognitive tasks by children aged 7–8 years.

Other prefrontal and motor cortex regions in the present study did not show significant changes in activation. One possible explanation is that cognitive changes are heavily dependent on the function of the prefrontal cortex, which may have contributed to the lack of significant changes before and after motor cortex activation. In addition, important subcortical areas, such as the hippocampus, are difficult to detect and could be considered a limitation of the present study.

## 5 Conclusion

In this study, 12-weeks of HRT were associated with may improve reaction time and accuracy on the Flanker task, suggesting a potential benefit of IC. In exploratory, unadjusted channel-wise fNIRS analyses, Oxy-Hb was increased in channels on approximately the right prefrontal region (putative R-DLPFC) under the congruent task. These findings are preliminary, task-specific, and do not demonstrate cognitive development or long-term functional improvement; larger samples and longer follow-up are needed to demonstrate this.

## 6 Limitations

Although we performed sample size calculations previously, the small sample sizes of the groups in this study may have limited statistical power, especially when multichannel fNIRS analysis was involved. Therefore, the results of this study should be interpreted with caution, and their generalizability may also be limited. Another limitation concerns diversity. The study examined multiple behavioral endpoints and exploratory channel fNIRS comparisons without formal correction for multiple comparisons. This increases the risk of false-positive findings; therefore, reported fNIRS channel results should be interpreted as hypothesis-generated and may no longer be significant after multiplicity correction. It is necessary to replicate in larger samples using a pre-specified correction procedure (e.g., FDR). In addition, given the very small sample size and the exploratory nature of this study, we did not report confidence intervals for behavioral effects, as interval estimates were unstable and might convey a misleading sense of precision; future confirmatory studies with sufficient power should report confidence intervals as well as multiple controlled effect estimates. Furthermore, follow-up was limited to immediate evaluation after the intervention and did not assess retention or long-term metastatic effects. Finally, horseback riding may induce greater novelty, motivation, and engagement compared with comparative interventions, which may affect behavioral performance and task-induced hemodynamic responses. Future studies with larger and more diverse samples, pre-specified multiple control procedures, and longer follow-up are needed to confirm the durability, generalizability, and mechanisms of these findings.

## Data Availability

The datasets generated and analyzed during the current study are not publicly available due to privacy and ethical restrictions but are available from the corresponding author on reasonable request.
